# Oncogene TUBA1C promotes migration and proliferation in hepatocellular carcinoma and predicts a poor prognosis

**DOI:** 10.18632/oncotarget.21894

**Published:** 2017-10-10

**Authors:** Ji Wang, Wei Chen, Weiwei Wei, Jianying Lou

**Affiliations:** ^1^ Department of Hepatobiliary and Pancreatic Surgery, The Second Affiliated Hospital of Zhejiang University, School of Medicine, Hangzhou, Zhejiang, P.R. China; ^2^ Department of General Surgery, Tongji Hospital, Tongji Medical College, Huazhong University of Science and Technology, Wuhan, Hubei, P.R. China

**Keywords:** prognosis, HCC, TUBA1C

## Abstract

The prognostic biomarkers and potential therapy targets are urgently needed in hepatocellular carcinoma (HCC). In this article, we report the expression of TUBA1C was significantly increased in HCC on mRNA and protein level, and this finding was further validated in another two independent datasets. Survival analysis was also implemented on these three datasets, and TUBA1C high expression group was detected to have relative shorter survival time. Furthermore, the metastatic ability is increased along with TUBA1C abundance, according to protein abundance evaluation of normal-tumor-portal vein tumor thrombus pairs, and mRNA comparison between metastasis-averse HCC and metastasis-incline HCC. Correlation analysis was implemented and TUBA1C expression was shown to be significantly associated with recurrence, embolus, and AFP level. Proliferation and migration assays following knock down of TUBA1C in two cell lines, HCCLM3 and PLC, revealed that down-regulation of TUBA1C significantly reduces proliferation and migration in HCC cells. *in vivo* study also showed the similar results. Gene Set Enrichment Analysis (GSEA) comparing the TUBA1C-low and TUBA1C-high group indicates that KEGG pathways including “cell cycle”, “DNA replication”, and “proteasome” were significantly enriched in TUBA1C-high group. In conclusion, prognostic biomarker and oncogene TUBA1C promotes migration and proliferation of hepatocellular carcinoma cells, probability via cell cycle signaling pathway.

## INTRODUCTION

Hepatocellular carcinoma (HCC) is the fifth leading cancer and the third causes of cancer related deaths [1]. The low five-year survival rate of HCC results from the fast progression and limited available drugs [2]. Thus, prognostic biomarkers and potential therapy targets are urgently needed to facilitate the prognosis and improve life quality of HCC patients.

Effort has been satisfied to investigate the prognostic molecular biomarkers and potential therapy targets. Among these biomarkers, tubulins were among the mostly reported gene associated with various cancer, including HCC. For instance, decreased expression of β III tubulin was reported to be associated with unfavorable prognosis in melanoma [3], and the similar function of this gene in prognosis was also reported in bladder cancer, ovarian cancer, renal cell carcinoma, and breast cancer [4–7]. Another tubulin, gamma-tubulin was reported to be associated survival of astrocytoma patients [8]. Increased α-tubulin1b expression has been shown to predict poor prognosis and resistance to chemotherapy in hepatocellular carcinoma [9].

In this article, we report that another oncogene, TUBA1C, as a component of tubulin, is significantly highly expressed in tumor tissues than the normal across datasets. The clinical significance of TUBA1C, including its association with clinical information, and its prognostic effect, was evaluated. Functional assays were performed to evaluate the metastasis and proliferation ability after knock down of TUBA1C *in vitro* and *in vivo*. Gene Set Enrichment Analysis (GSEA) comparing high/low TUBA1C expression group indicates that KEGG pathways including “cell cycle”, “DNA replication”, and “proteasome” were significantly enriched.

## RESULTS

### TUBA1C expression is enhanced in HCC tissues

**Figure 1:**
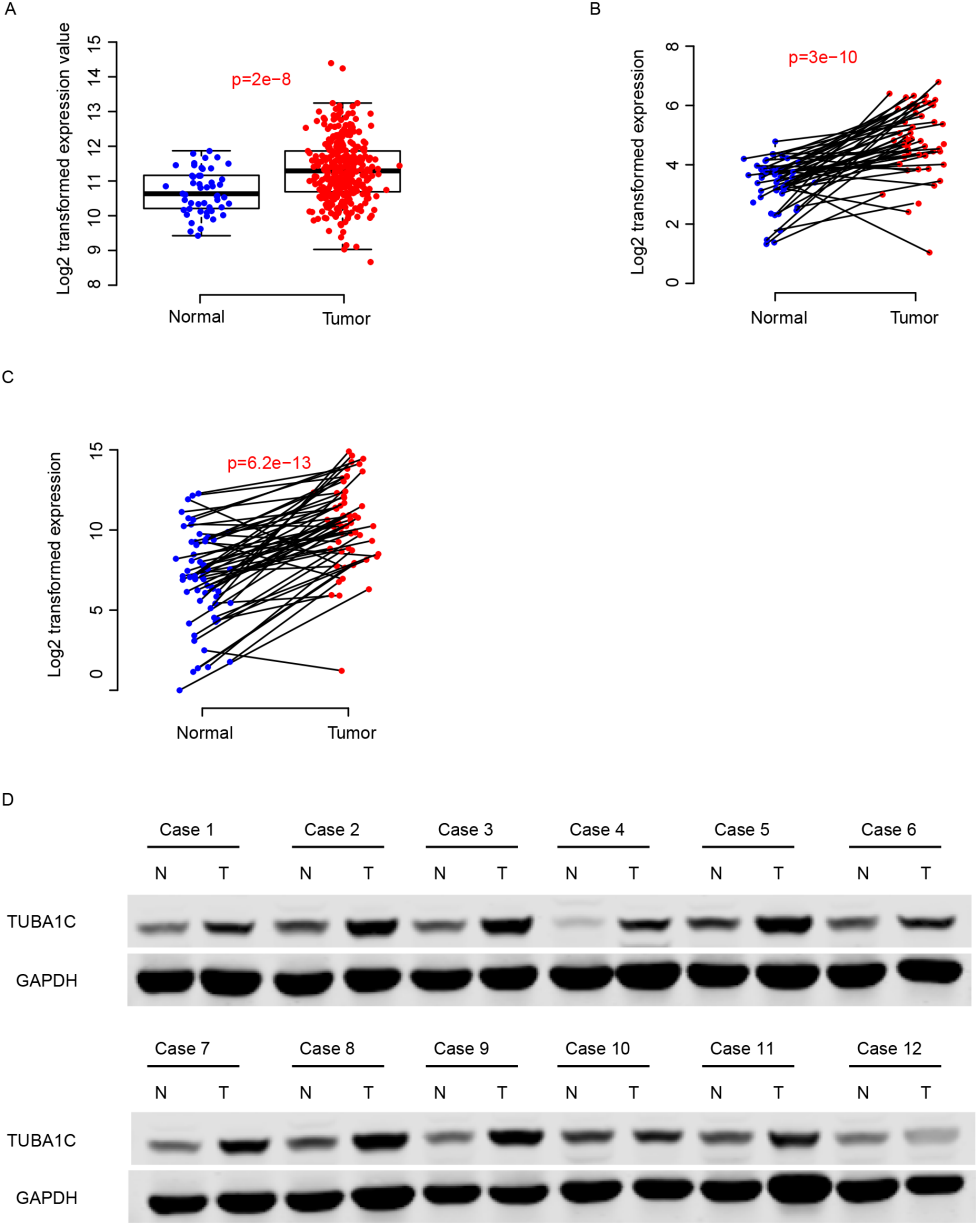
TUBA1C was up-regulated in hepatocellular carcinoma The TUBA1C mRNA was up-regulated in TCGA, GEO and QPCR dataset **(A-C**, respectively). The protein abundance of normal and tumor tissues showed similar results **(D)**. N indicates normal tissue and T refers to tumor tissue.

Expression values of TUBA1C of normal and cancerous tissues were compared in two independent datasets, TCGA-LIHC and GEO dataset (GSE77314). The expression of TUBA1C was significantly enhanced in tumor tissues compared to the adjacent normal tissues (Figure 1a-1b). Furthermore, using q-RTPCR, the expression values normal and tumor tissues was also quantified for validation, and the result resembles the pattern in the aforementioned datasets (Figure 1c). The protein abundance of TUBA1C in the tumor tissues was also significantly higher than adjacent normal tissues, according to Western Blot results (Figure 1d). All these results above indicate that TUBA1C was up-regulated in HCC tissues.

### TUBA1C is a prognostic biomarker for HCC

**Figure 2:**
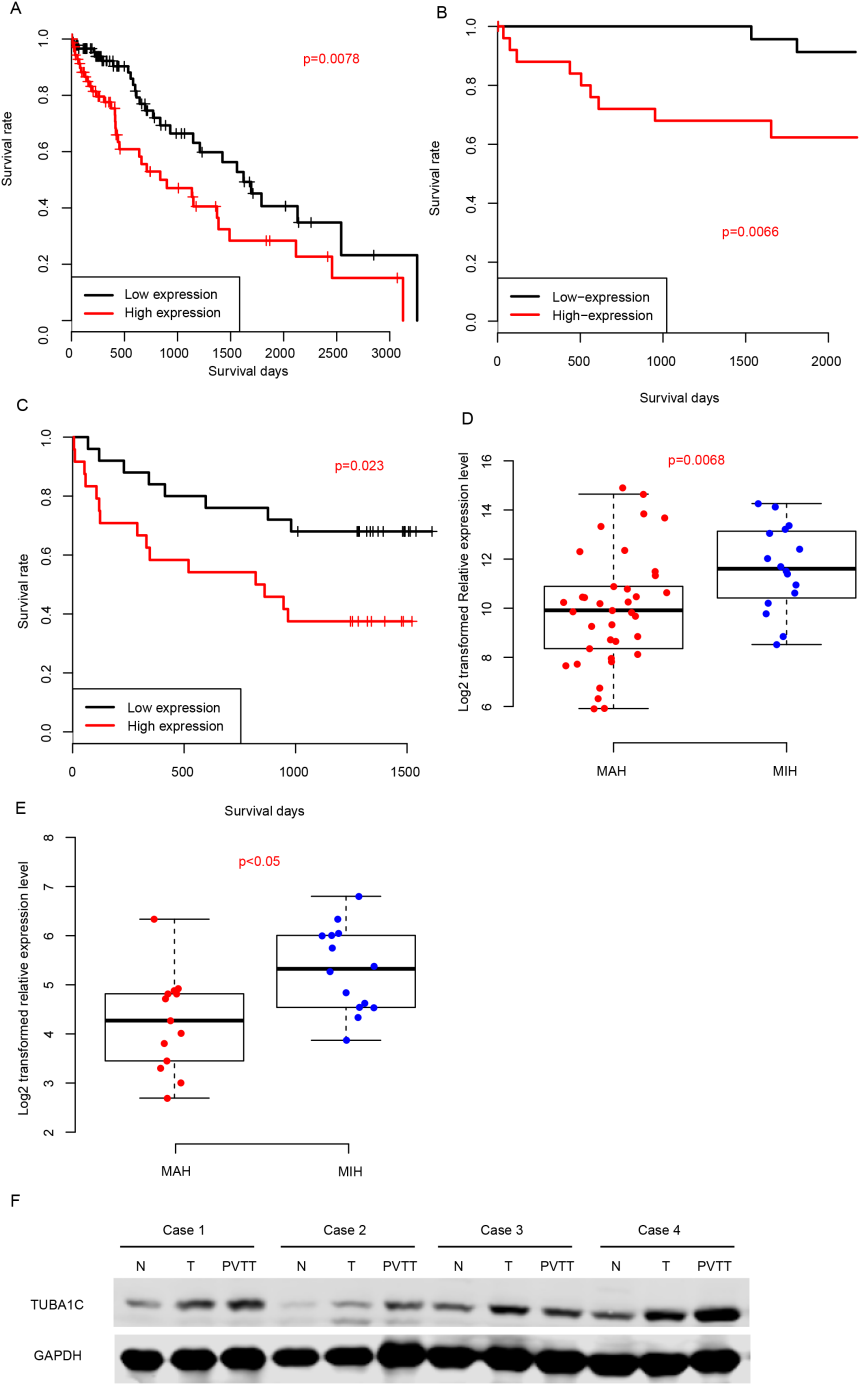
Prognostic effect of TUBA1C Correlation between TUBA1C expression and survival in TCGA **(A)**, GEO **(B)** and QPCR **(C)** datasets. TUBA1C expression values in MIH and MAH group were compared in QPCR **(D)** and GEO **(E)** datasets. The y-axis indicates log 2 transformed TUBA1C expression level. Protein abundance of TUBA1C in normal-Tumor-PVTT pairs **(F)**.

We also evaluated the prognostic effect of TUBA1C by dividing the samples into TUBA1C-high and TUBA1C-low group according to its median expression value in TCGA-LIHC, GSE77314 and q-RTPCR datasets (Figure 2A-2C). The TUBA1C-high group had a significantly shorter survival time than TUBA1C-low group in these three datasets. In addition, we classified the samples into metastasis-averse HCC (MAH) and metastasis-incline HCC (MIH) based on the clinicopathological indicators and follow up information, and compared the relative expression values of TUBA1C in these groups. As expected, the TUBA1C expression in MIH group is significantly higher than MAH group in two aforementioned datasets (Figure 2D-2E). Portal vein tumor thrombus (PVTT) are cancer cells migrate from primary tumor tissue to portal vein, thus cells in PVTT have more migration ability than the resident tumors. We then compared the protein abundance in normal-tumor-PVTT pairs, and the results showed that PVTT had a significantly higher protein abundance of TUBA1C than the primary tumor tissue (Figure 2F). In summary, high expression of TUBA1C is associated with metastasis, and predicts a poor survival.

### Clinicopathological indicators and TUBA1C

**Figure 3:**
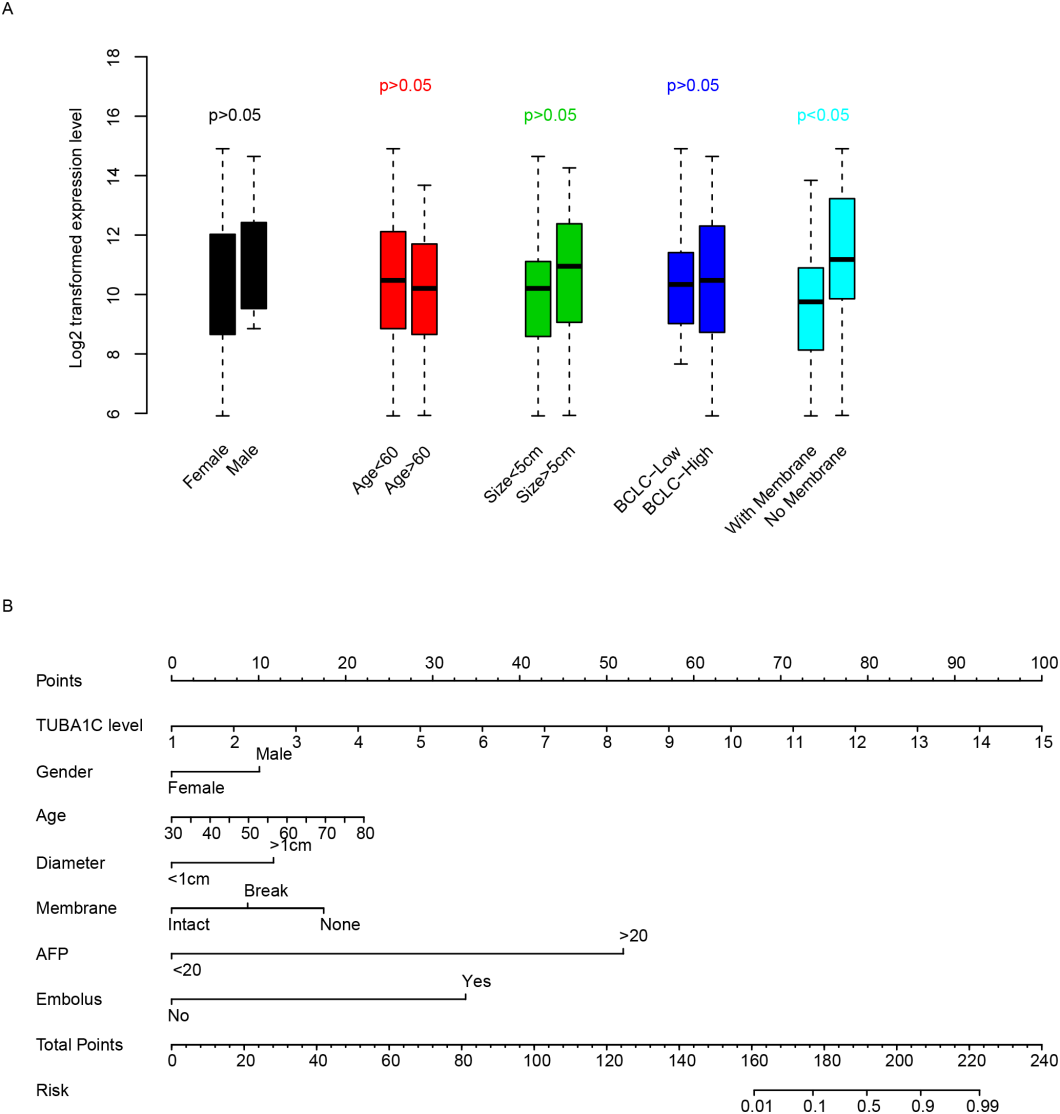
Clinical effect of TUBA1C TUBA1C expression in clinical categories was compared **(A)**. The y-axis indicates log 2 transformed TUBA1C expression level. A nomogram using clinical information and TUBA1C expression was plotted **(B)** to predict three-year survival rate. The clinical indicators were used to calculate the risk points and the summed risk points was used to evaluate the risk of events (death within three years.)

The correlation between clinicopathological and TUBA1C expression was evaluated in qPCR dataset (Table 1). To facilitate the comparison, we artificially divided patients into TUBA1C-high and TUBA1C-low group based on the median expression level of TUBA1C, as usual. Among these indicators, we noted that TUBA1C is significantly associated with recurrence, 42.11% (8/19) patients in TUBA1C-high group relapsed, while only 10% (2/20) samples in TUBA1C-low group detected recurrence (p=0.0310). TUBA expression was also shown to be associated with tumor embolus, with 35.71% (10/28) in TUBA1C-high group and 10.34% (3/29) in the TUBA1C-low group (p=0.0295). In addition, high level of AFP (alpha-fetoprotein) was also detected to be associated with TUBA1C expression. Besides, we noticed that TUBA1C was independent from gender, age, tumor size and differentiation, while it is significantly associated with membrane status (Figure 3a). To facilitate the utilization of TUBA1C for prognosis, a nomogram considering age, gender, tumor size, membrane status, AFP and embolus was plotted to predict three-year survival rate of HCC patients (Figure 3b). It was noticed that the TUBA1C expression level contributed more risk score to the prognosis of HCC (ranged 0-100), indicating the importance of this gene for prognosis compared to other clinical indicators.

**Table 1:** Correlation between clinical observations and TUBA1C

Variables	TUBA1C-Low	TUBA1C-High	pvalue
**Reccurence**			**0.031**
No	18	11	
Yes	2	8	
**differentiate**			0.103
1-2	9	3	
3-4	20	25	
**Primary Tumor stage**			0.056
1-2	26	19	
3-4	3	9	
**Membrane**			0.171
No	19	11	
Yes	6	8	
**daughter_nodule**			0.079
No	27	21	
Yes	2	7	
**embolus**			**0.029**
No	26	18	
Yes	3	10	
**AFP**			**0.014**
Low(<20)	16	7	
High(>20)	11	22	

### TUBA1C promotes proliferation and migration in HCC cell lines

**Figure 4:**
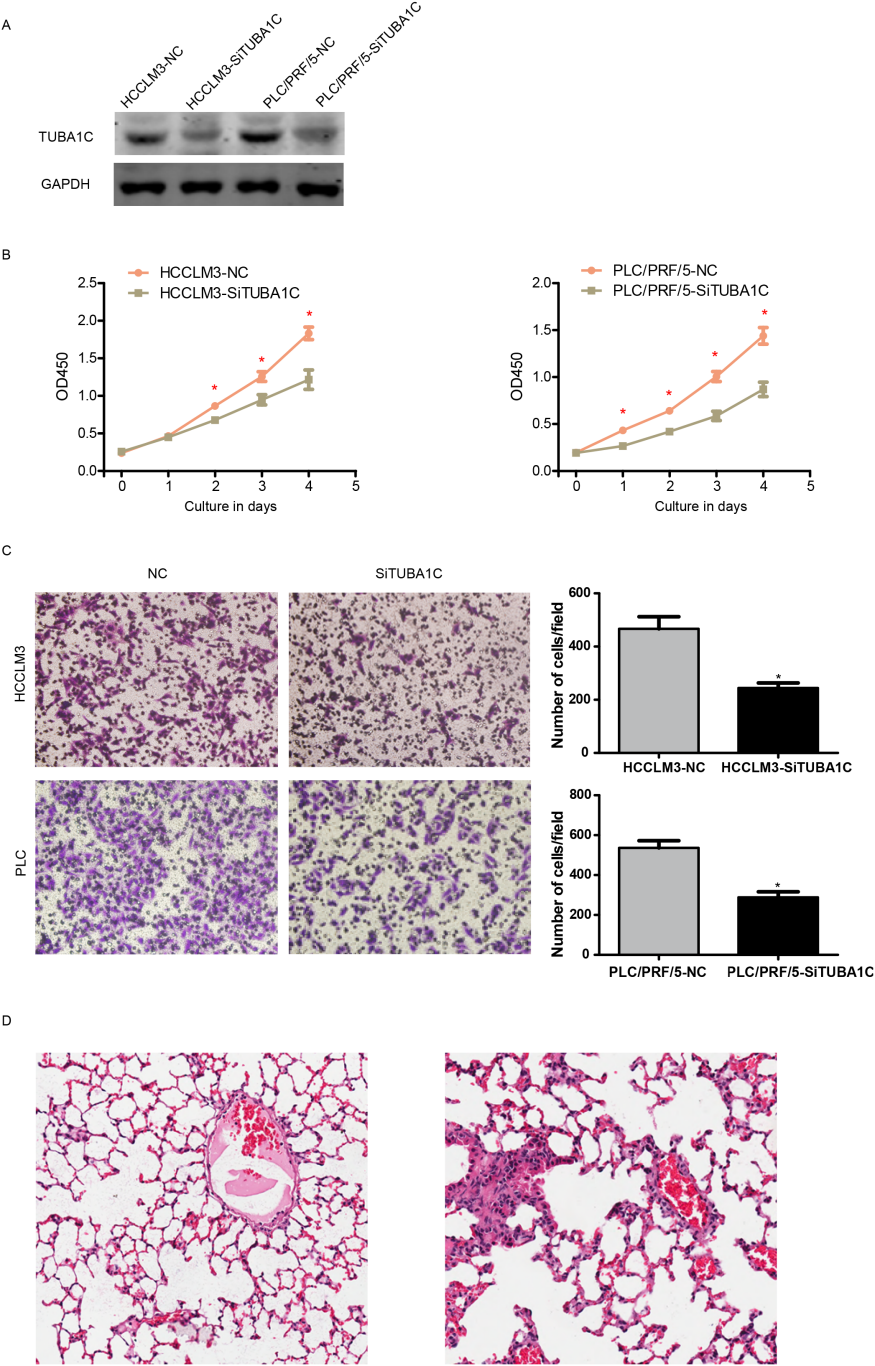
TUBA1C promotes metastasis and proliferation *in vitro* and *in vivo* After knocking down of TUBA1C **(A)**, the proliferation rate of HCC cells was decreased in both HCCLM3 (**B**, left) and PLC/PRF/5 cell line (B, right). The migration rate was also decreased both *in vitro* **(C)** and *in vivo* **(D**, left, non-metastatic TUBA1C knock down group, right metastatic control group).

To validate the fact TUBA1C associated with migration, we tested the proliferation migration ability of two different HCC cell lines, PLC and HCCLM3, following TUBA1C knock down using siRNAs (Figure 4A). Cell concentration was evaluated using CCK8 kit every 24 hours, and the result show that the proliferation rate of HCC cells was significantly reduced after TUBA1C knock down in both cell lines (Figure 4B).

The migration rate was also measured following TUBA1C knock down using the same method. After incubation for 14 hours, the migrated cell number was compared (Figure 4B). Migrated cells in the YUBA1C knock down group were significantly less than the control group, in both PLC and HCCLM3 cell lines (Figure 4C). *in vivo* migration assay was also implemented. After rejection of TUBA1C overexpressed cell line (termed HCCLM3-TUBA1C) and control group (HCCLM3-GFP), the lung metastasis was identified after house for 12 weeks (Figure 4D). Four out six mouse injected HCCLM3-TUBA1C and one out of six in HCC-GFP group was detected to have metastatic loci in in the lung. All these results indicate that aberrant expression of TUBA1C is associated with migration and proliferation of HCC cells, both *in vivo* and *in vitro*.

### Pathways associated with TUBA1C expression

**Figure 5:**
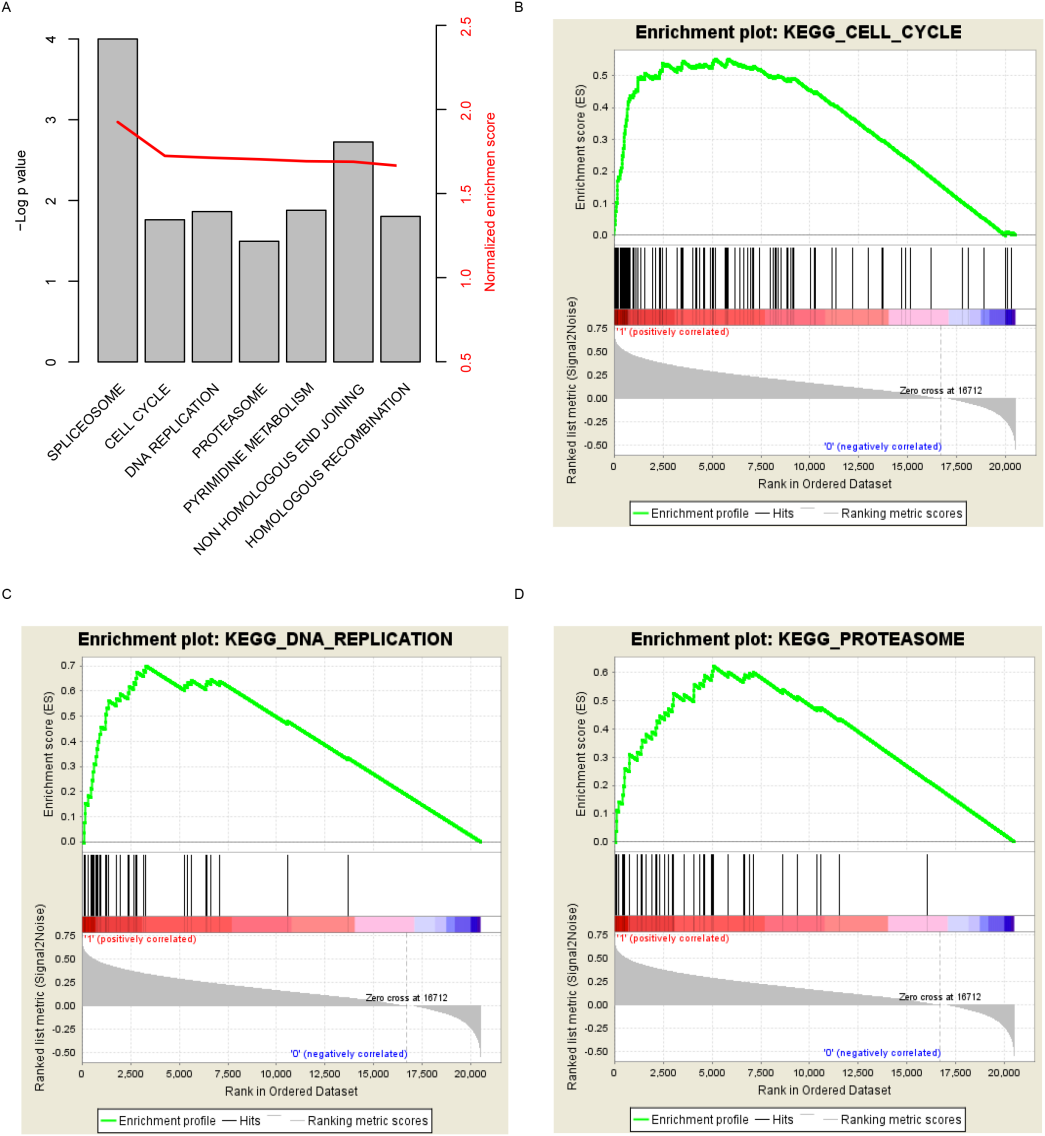
KEGG pathways associated with TUBA1C expression Significantly enriched KEGG pathways **(A)**, including cell cycle **(B)**, DNA replication **(C)** and proteasome **(D)**.

In order to investigate the pathways that TUBA1C may regulate or effect, Gene Set Enrichment Analysis was carried out by comparing expression of genes in the TUBA1C-high/low group based on transcriptome of samples provided in TCGA. Curated KEGG pathways was used in this step. Among these pathways, carcinogenesis and development associated (Figure 5A), including “cell cycle”, “DNA replication”, and “proteasome” were identified as significantly altered along with aberrant TUBA1C expression (Figure 5B-5D). We noted that genes involved in cell cycle signaling pathway was significantly altered in TUBA1C-high group, which may explain the high proliferation rate. In summary, TUBA1C expression alters prognosis of hepatocellular carcinoma may via cell cycle signaling pathway.

## DISCUSSION

Lack of reliable potential prognostic biomarkers and therapy target makes HCC prognosis and treatment difficult. In current work, by analyzing the expression of TUBA1C in HCC, we investigated the prognostic effect of TUBA1C, studied the function of TUBA1C on proliferation and migration. The results indicate that TUBA1C is a potential diagnostic and prognostic biomarker for HCC, and aberrant expression of TUBA1C significantly altered the proliferation and migration ability of HCC cell lines. Potential pathways associated with TUBA1C expression includes cell cycle, DNA replication, and proteasome. All these results indicate that proteasome is a potential biomarker for HCC prognosis.

TUBA1C was known as encoding an alpha tubulin, associated with microtubule formation [10] and involved in microtubule based cell processes, including cytoskeleton-dependent intracellular transport, and cell division. Another report has shown that TUBA1C encoded 42KD protein abundance was evaluated in HCC, compared to the adjacent normal tissues in well differentiated HCV-related HCC. However, other reports regarding the function of this gene is still limited, hitherto. We have observed that the expression of TUBA1C was associated with pathways including cell cycle, which is consistent with previous report. Since the function of TUBA1C is a component of tubulin, we suspect that this gene may participate in cell spindle formation and cell junction, and thus involved in cell proliferation and migration. The GSEA pathway analysis and function assay results was consistent with this hypothesis.

In summary, in this article, as a novel prognostic biomarker for HCC prognosis, TUBA1C regulated cell proliferation and migration, via cell cycle signaling pathway.

## MATERIALS AND METHODS

### qRT-QPCR quantifies TUBA1C

All patients involved in this study have provided written inform consent and this study has been approved by The Second Affiliated Hospital of Zhejiang University Ethnic Committee. Total RNA from cancer and normal tissues was isolated with Trizol (Invitrogen, CA) using the guiding manual. Quantity and quality of RNA was assessed with Nanodrop 2000 (Thermo Scientific, USA). Random primers and M-MLV Reverse Transcriptase (Invitrogen, CA) was used to synthesize the first strand cDNA from 3μg total RNA. Quantification of TUBA1C use real-time polymerase chain reaction (RT-PCR) with SYBR Green (Applied TaKaRa, JA) according to the manufacture provided protocols. Normalize the relative expression values from different batches with endogenous control, 18S RNA, and relative CT values. Each sample involved were tested in duplicate, and mean values were calculated for further analysis.

### Cell culture and transfection

Two different hepatocellular carcinoma cell lines, HCCLM3 and PLC, were purchased from Cell Bank of Type Culture Collection of Chinese Academy of Sciences. All HCC cell lines were cultured in the incubator with the following conditions: 5% CO2, temperature 37°C, in Dulbecco's modified Eagle's medium supplementing 10% fetal bovine serum. For the TUBA1C knocking down, two distinct siRNAs (Biotend, Shanghai, People's Republic of China) against TUBA1C (sequences: 5’UGACCUUGUUGUGGUCCAGdTdT and 5’UUAUUGGUAAGGGCGAGGGdTdT) was transfected into PLC and HCCLM3 cell lines following manufacture provided user manual, and the efficiency of siRNAs was evaluated.

### Western blot

Protein extraction was implemented with RIPA Lysis Buffer based on the manufacturer provided manual, and then centrifuged at 12,000 rpm for 15 minutes. The total protein concentration of samples was evaluated with the standard bicinchoninic acid assay. The TUBA1C antibody (Abcam, Shanghai, China) was diluted at 1:500, and endogenous control, GAPDH was diluted at 1:10000, (Santa Cruz Biotechnology). Immuno-complexes were further incubated with the fluorescein-conjugated secondary antibody. The antibody binding signal intensity was scanned and analyzed with Odyssey infrared scanner (Li-CorBiosciences, Inc.)

### Migration and proliferation assay *in vivo* and *in vitro*

Transwell filter chambers to was used assess the migration ability of HCC cell lines (Costar, Corning, NY) based on the protocols provided by manufacturer. Re-suspend ~1×10^5^ cells in serum-free medium, gently shock several times, add into the top of the chamber, and add medium containing 10% FBS into the opposite side of chamber. Culture the cells for 14 hours, stain, photograph, and count the cells on the lower surface of the membrane using a microscope in three random fields per field for each assay. Each step was performed three times. For the cell proliferation assay, HCC cells were seeded into a 96-well plates (3000/well) and use Cell Counting Kit-8 (Dojindo Laboratories, JA) per 24 hours, following the manufacturer provided protocols to test the proliferation rate. Twelve 6-week-old male nude mice were randomized into two groups (N=6, for each group). HCCLM3-TUBA1C and HCCLM3-GFP cells (5×10^5^) were injected into the tail vein of these mouse. Mice was sacrificed at 18 weeks after injection and lungs were dissected and H&E staining. Animals were housed in cages under standard conditions, following the guideline of the The Second Affiliated Hospital of Zhejiang University and the National Institutes of Health.

### Statistical analysis

All data analysis and graphing was performed with R and R packages. The receiving operating characteristic curve was plotted and calculated with R package “pROC”[11]. Survival analyses were implemented with R package “survival”, and Gene Set Enrichment analysis was carried out with GSEA java software [12]. Correlation between TUBA1C expression and clinical information was evaluated with fisher's exact test. The TUBA1C-high and TUBA1C-low expression group was defined by the median expression value of TUBA1C, as cutoff.
